# The Pan-American Medical Congress

**Published:** 1891-11

**Authors:** 


### BUFFALO MEDICAL AND SURGICAL JOURNAL

A MONTHLY REVIEW OF MEDICINE AND SURGERY.

*EDITORS:*

THOMAS LOTHROP, M. D. - - WM. WARREN POTTER, M. D.

All communications, whether of a literary or business character, should be addressed to the managing editor : 284 Franklin Street, Buffalo, N. Y.

Vol. XXXI. NOVEMBER, 1891. No. 4.

### THE PAN-AMERICAN MEDICAL CONGRESS.

Our readers are undoubtedly familiar with the fact, that at the last meeting of the American Medical Association, held in Washington, May, 1891, a committee, consisting of one member from each State and Territory, was appointed to organize a medical congress, which should be composed of delegates and members from the several countries comprising the Western Hemisphere. This committee met in the city of St. Louis, October 14, 15, and 16, 1891, and perfected the organization of the proposed medical congress.

Dr. Chas. A. L. Reed, Chairman, called the committee to order at four o'clock on the 14th, and it continued its work during the 15th and 16th, until the organization was finally perfected. Washington was chosen as the place of meeting, and the date fixed upon was the first Tuesday of October, 1893.

Dr. William Pepper, of Philadelphia, was elected President, and Dr. Chas. A. L. Reed, of Cincinnati, was chosen for the important office of Secretary-General. To Dr. Reed is due the conception and inauguration of the systematic scheme and extensive correspondence with professional men, not only in the United States and Canada, but in the South American Republics, which has finally resulted in organizing the first Pan-American Medical Congress ever proposed or held. It was a graceful act on the part of the committee to select Dr. Reed for the important post of Secretary-General, and it may be affirmed that no man in this country is more familiar with the duties pertaining to that office than the accomplished Cincinnatian who has been selected to fill it.

There are so many details connected with the organization of such a congress, that it is highly important that a man of great executive capacity should be at the helm. Dr. Pepper's talent in the direction named is well known, and he is a conspicuous American physician of great and versatile resource. With Dr. Pepper and Dr. Reed pulling together, like a double team in well and easily fitting harness, as they no doubt will, it may be assumed that the first Pan-American Medical Congress is already an assured success. The remainder of the organization, such as vice-presidents and officers of sections, will be duly announced, and, no doubt, the journals will soon give the full list. Let the profession and the government of the United States yield cordial support to a grand enterprise so auspiciously inaugurated.

